# Novel Roles for K_v_7 Channels in Shaping Histamine-Induced Contractions and Bradykinin-Dependent Relaxations in Pig Coronary Arteries

**DOI:** 10.1371/journal.pone.0148569

**Published:** 2016-02-04

**Authors:** Xingjuan Chen, Wennan Li, S. Christopher Hiett, Alexander G. Obukhov

**Affiliations:** Department of Cellular and Integrative Physiology, Indiana University School of Medicine - Indianapolis, Indianapolis, Indiana, 46202, United States of America; University at Buffalo, UNITED STATES

## Abstract

Voltage-gated K_v_7 channels are inhibited by agonists of G_q_-protein-coupled receptors, such as histamine. Recent works have provided evidence that inhibition of vascular K_v_7 channels may trigger vessel contractions. In this study, we investigated how K_v_7 activity modulates the histamine-induced contractions in “healthy” and metabolic syndrome (MetS) pig right coronary arteries (CAs). We performed isometric tension and immunohistochemical studies with domestic, lean Ossabaw, and MetS Ossabaw pig CAs. We found that neither the K_v_7.2/K_v_7.4/K_v_7.5 activator ML213 nor the general K_v_7 inhibitor XE991 altered the tension of CA rings under preload, indicating that vascular K_v_7 channels are likely inactive in the preloaded rings. Conversely, ML213 potently dilated histamine-pre-contracted CAs, suggesting that K_v_7 channels are activated during histamine applications and yet partially inhibited by histamine. Immunohistochemistry analysis revealed strong K_v_7.4 immunostaining in the medial and intimal layers of the CA wall, whereas K_v_7.5 immunostaining intensity was strong in the intimal but weak in the medial layers. The medial K_v_7 immunostaining was significantly weaker in MetS Ossabaw CAs as compared to lean Ossabaw or domestic CAs. Consistently, histamine-pre-contracted MetS Ossabaw CAs exhibited attenuated ML213-dependent dilations. In domestic pig CAs, where medial K_v_7 immunostaining intensity was stronger, histamine-induced contractions spontaneously decayed to ~31% of the peak amplitude within 4 minutes. Oppositely, in Ossabaw CAs, where K_v_7 immunostaining intensity was weaker, the histamine-induced contractions were more sustained. XE991 pretreatment significantly slowed the decay rate of histamine-induced contractions in domestic CAs, supporting the hypothesis that increased K_v_7 activity correlates with a faster rate of histamine-induced contraction decay. Alternatively, XE991 significantly decreased the amplitude of bradykinin-dependent dilations in pre-contracted CAs. We propose that in CAs, a decreased expression or a loss of function of K_v_7 channels may lead to sustained histamine-induced contractions and reduced endothelium-dependent relaxation, both risk factors for coronary spasm.

## Introduction

In the coronary circulation, histamine is a local hormone, or autacoid that is released from the mast cells [1] infiltrating the atherosclerotic segments of coronary artery wall [2]. The histamine H1 G_q_-protein-coupled receptor is highly expressed in both “healthy” and atherosclerotic metabolic syndrome (MetS) coronary arteries [3–5]. Activation of the histamine H1 receptors results in potent contractions of both pig and human coronary arteries [4,6,7]; and contractile responses to histamine are more pronounced in severely atherosclerotic segments of human coronary arteries [8]. Therefore, it is not surprising that elevated blood plasma concentration of histamine has been linked to poor prognosis in patients suffering from ischemic heart diseases, such as stable coronary artery disease and acute coronary syndrome [6,9].

K_v_7 potassium channels are activated by cell membrane depolarization. Uniquely, these channels can be inhibited by the agonists of the G_q/11_-protein-coupled receptors, including histamine [10]. The K_v_7 subfamily consists of five members (K_v_7.1- K_v_7.5) and expression of these isoforms varies among cell types. Earlier studies showed that K_v_7.1 channels are highly expressed in the heart [11,12], whereas the K_v_7.2 and K_v_7.3 channels are predominantly expressed in the nervous system. Recently, it has been shown that K_v_7.4 and K_v_7.5 channels are primarily expressed in vascular smooth muscle cells [13–15]. Physiologically, K_v_7 activity contributes to the hyperpolarization of the smooth muscle cell membrane that limits the activation of voltage-gated calcium channels, which are known to precipitate vasoconstriction [16]. A significantly lower expression of K_v_7.4 has been reported in the renal, mesenteric, and coronary arteries from several rodent hypertensive models [17,18], suggesting that the channel is important for regulating vascular tone. Indeed, nonselective K_v_7 channel blockers, XE991 and linopirdine, can induce vasoconstriction by producing membrane depolarization and enhancing calcium influx through voltage-gated calcium channels in some vascular beds, such as mesenteric and pulmonary arteries [14,19–21]. On the other hand, K_v_7 activators are reported to induce relaxation of pre-contracted arteries by hyperpolarizing the smooth muscle cell membrane potential [22]. However, the functional implication of K_v_7 inhibition and activation in coronary artery responses to vasoconstrictive hormones, such as histamine, is not fully elucidated, specifically in the setting of MetS, a risk factor for coronary artery disease.

In the present study, we investigated the distribution pattern of K_v_7 proteins in the wall of “healthy” and MetS pig right coronary arteries (CAs). We also explored the effects of endothelial and smooth muscle K_v_7 activation and inhibition on the reactivity of “healthy” and MetS RCAs in the presence and absence of histamine utilizing an *ex vivo* pig CA ring model.

## Materials and Methods

### Animals

All of the animal experiments were approved by the Indiana University School of Medicine Institutional Animal Care and Use Committee and strictly adhered to the guidelines described in the Guide for the Care and Use of Laboratory Animals published by the United States National Institutes of Health. 8–20 month-old domestic and Ossabaw pigs (50–100 kg) were used (n = 31 pigs, both males and females). The animals were anaesthetized by using a mixture of 100 mg/ml telazol, 50 mg/ml ketamine, and 50 mg/ml xylazine (0.044 ml per kg, intramuscularly), followed by isoflurane inhaled via an endotracheal tube. Neuromuscular blocking agents were not used. The anaesthetized pigs were euthanized by removing the heart. Some domestic pig right CAs were provided to us by other investigators (Drs. Jose Estrada, Mouhamad Alloosh, Michael Sturek, Adam Goodwill, and Johnathan D. Tune).

### Ossabaw Pigs Model of MetS

The diet-induced MetS Ossabaw pig model was utilized in this study. Six month old pigs were divided into two groups. The lean pig group was kept on the standard feed with 22% of kilocalories from protein, 70% from carbohydrates, and 8% from fat. The MetS pig group was fed a high-fat diet containing 13% of kilocalories from protein, 40% from carbohydrates, and 47% from fat supplemented with 2% cholesterol. The development of MetS required about 7 months. The detailed information on characterization and development of the pig model of MetS is provided in [23].

### Isometric tension recordings

The domestic pig CAs were isolated and cleaned from the connective tissue and fat. The right CAs (RCAs) with a diameter of about 1–2 mm were cut into 2–3 mm rings. The isometric tension experiments were performed as described previously [24,25]. Briefly, the rings were mounted into organ baths containing oxygenated (saturated with a gas mixture of 95% O_2_ and 5% CO_2_) Krebs buffer that was maintained at 37°C. The Krebs buffer contained (mM): 131.5 NaCl, 5 KCl, 2.5 CaCl_2_, 1.2 NaH_2_PO_4_, 1.2 MgCl_2_, 25 NaHCO_3_, and 10 glucose. The pH of 5% CO_2_-saturated solution had a value of ~7.4. The CA ring pre-load was set to 2–3 g. All of the experiments related to the role of K_v_7 channels in the endothelium were performed in the presence of 10 μM indomethacin to reduce the contribution of endogenous vasoconstrictive prostanoids that may be released by endothelial cells. A GlobalTown Microtechnology (Sarasota, FL) wire myograph was used to record vessel tensions.

### Immunohistochemistry

Coronary artery segments of 1 to 2 mm from domestic or Ossabaw pigs were formalin-fixed, paraffin embedded, and cross-sectioned. The sections were deparaffinized with xylene, hydrated, and subjected to the standard acidic antigen retrieval procedure. The primary goat K_v_7.4 antibody (dilution factor: 1:100, Santa Cruz Biotechnology, Dallas, TX) or rabbit K_v_7.5 antibody (dilution factor: 1:50, Alomone Labs, Jerusalem, Israel) were added to the prepared sections. After overnight incubation at 4°C with the corresponding primary antibody, the sections were then incubated with the secondary biotin-conjugated antibody (dilution factor 1:1,000, The Jackson Laboratory, Sacramento, CA). After the secondary antibody was removed by multiple washes, the sections were treated with horseradish peroxidase-conjugated to streptavidin. The color was developed using diaminobenzidine and appeared brown. The color density of staining was analyzed using the DAB analysis module of Image-Pro Premier 9.1 (Media Cybernetics Inc., Warrendale, PA). The ratio of the background-corrected intensities of media/intimal layer and the adventitia layer staining was calculated to quantify the relative expression levels of K_v_7 proteins. No staining was observed in “no primary antibody” controls (S1 Fig).

### Chemicals

Histamine was purchased from Sigma-Aldrich (St. Louis, MO). ML213, UCL2077, diclofenac, Prostaglandin F_2α_, bradykinin and XE991 were purchased from Cayman Chemical (Ann Arbor, MI).

### Data analysis and statistics

The SigmaPlot 12.5 software (Systat Software Inc., San Jose, CA) analysis module was used for performing all of the statistical analyses. The t-test followed by the Mann-Whitney Rank Sum Test or paired t-test was used to determine whether there is a statistically significant difference between two groups. The significance level was set to 0.05. All of the data were presented as mean±SEM. Each set of experiments was repeated 3 to 12 times, using artery segments isolated from 3 to 12 pigs. In figures, one asterisk (*) corresponds to p<0.05; two asterisks (**) mean p<0.001; three asterisks (***) indicate p<0.0001; and four asterisks (****) denote p<0.00001.

## Results

### K_v_7 channel modulators do not affect the tone in domestic pig CA preloaded rings

**Fig 1:**
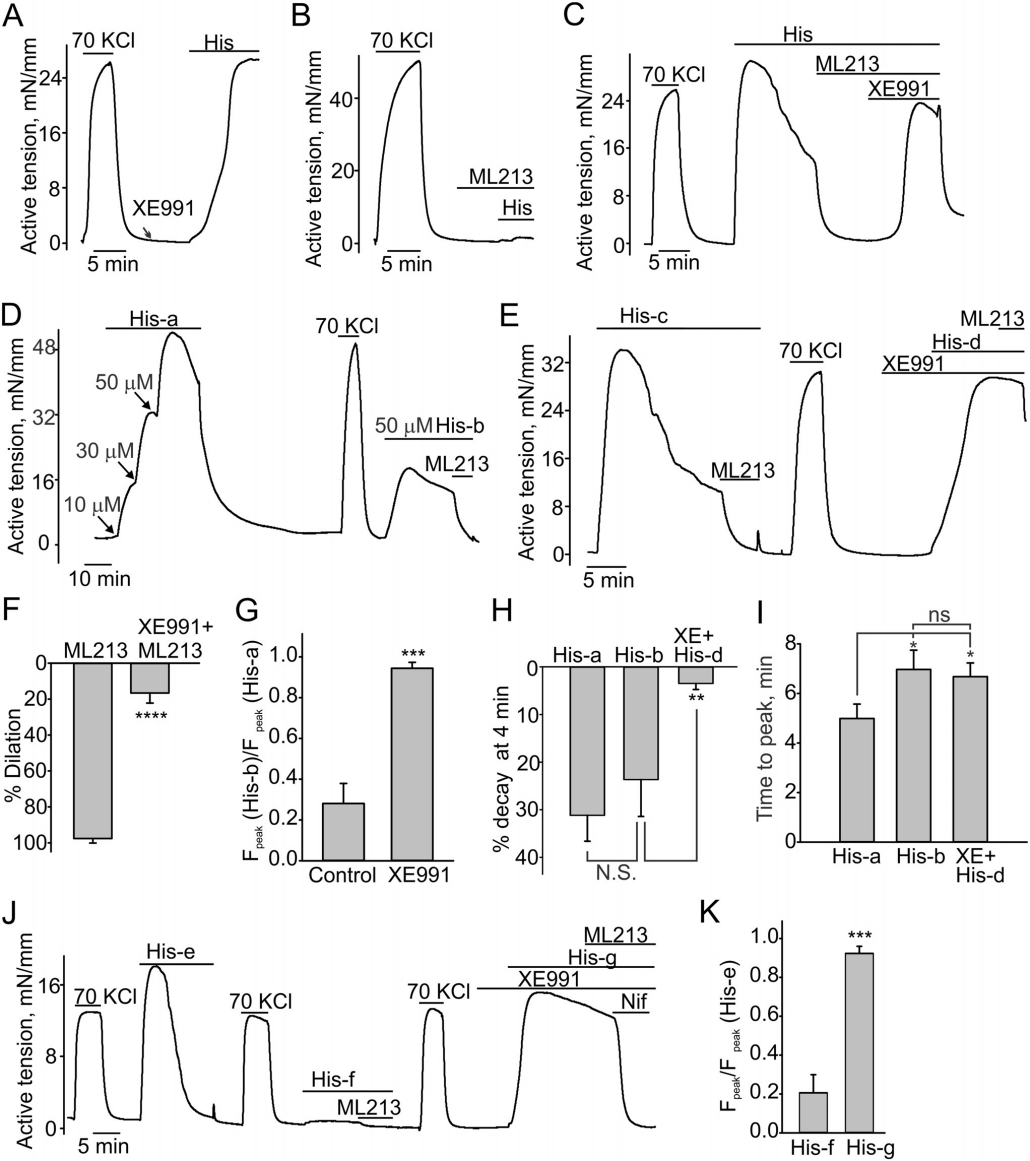
Effects of K_v_7 modulators on pig CAs. Sample isometric tension measurement traces and statistical analysis data are shown. 70 mM KCl-induced contractions were regularly assessed to monitor the general reactivity of the tested CA rings. Neither 10 μM XE991 (**A,** n = 6) nor 10 μM ML213 (**B,** n = 6) affected the 2–3 g preloaded CA ring tension. **C**, 10 μM ML213 potently dilated the pig CA pre-contracted with histamine (50 μM), whereas XE991 (10 μM) reversed the dilatory effect of ML213 (n = 4). **D**. Histamine concentration-dependently induced CA contractions. The shown isometric tension recording represents a time-matched control for “E.” The second histamine application induced a contractions with a smaller amplitude and slower onset kinetics (n = 4). **E** shows that the histamine-induced contractions exhibit slower kinetics of decay in the presence of 10 μM XE991 (n = 8). **F,** Comparison of 10 μM ML213-induced dilations of histamine-pre-contracted CA rings in the presence and the absence of 10 μM XE991. **G.** Comparison of normalized forces of contractions (F_peak_) in the presence and absence of 10 μM XE991 (summary data for “D” and “E”). The maximal force of the second histamine-induced contraction was normalized to the maximal force of the first histamine-induced contraction. **H**. Comparison of decay rates of histamine-induced contractions in the absence and presence of 10 μM XE991. **I**. Comparison of time to peak values for the indicated groups. **J**. A sample trace illustrating that 10 μM XE991 can completely restore the magnitude of histamine-induced contractions even when there was no apparent contractile response to a preceding histamine application. 10 μM nifedipine was added to the bath to assess the contribution of voltage-gated Ca^2+^ channels in mediating the contraction. **K**. Summary of data that are shown in “J” (n = 4).

Previous works by other groups showed that K_v_7 channels exhibit a threshold of activation ranging from -62 mV to -50 mV [26,27] and a half activation voltage of around -8 mV for K_v_7.1 [26], -12 mV for K_v_7.2 [28], -30 mV for K_v_7.3 [28], -13 mV for K_v_7.4 [28], and -47 mV for K_v_7.5 [29]. Moreover, Liu et al. (2008) demonstrated [10] that histamine inhibits at least the K_v_7.2 channel activity. On the other hand, Morales-Cano et al. (2015) found that K_v_7 channels are active in the resting rat CAs [30]. If K_v_7 channels are also active in preloaded pig CAs, then inhibition of the channel activity by histamine may lead to coronary artery smooth muscle cell depolarization. This would first cause voltage-gated Ca^2+^ channel activation followed by CA contraction. Therefore, we hypothesized that K_v_7 channel inhibition by histamine or a small molecule blocker would be sufficient to trigger CA contractions. To test this hypothesis, we performed isometric tension studies. We first investigated if a general K_v_7 inhibitor XE991 causes CA contractions. Unexpectedly, we found that XE991 did not significantly affect preloaded ring tension (Fig 1A, 1E and 1J). We next tested if the K_v_7.2/7.4/7.5 activator ML213 (Fig 1B) would dilate 2–3 g preloaded CA rings. But again, no significant changes in ring tone were observed during these experiments as well. These findings indicate that K_v_7 channels are likely inactive or negligibly active in the preloaded CA. Interestingly, while XE991 did not impede histamine-induced contractions, ML213 effectively prevented those almost completely (Fig 1A and 1B).

### XE991 and ML213 modulate histamine-induced contractions in domestic CA rings

We next investigated whether K_v_7 modulators affect either the amplitude or kinetics of histamine-induced contractions. We first challenged the rings with ML213 and found that the K_v_7 activator potently dilated histamine-pre-contracted domestic CA rings (Fig 1C, 97.6±2.05% of dilation, n = 4) with an EC_50_ of about 2.5 μM. The K_v_7 inhibitor, XE991, reversed the ML213-induced relaxations in histamine-pre-contracted domestic CA rings (Fig 1C). XE991 also prevented the ML213-induced dilations in histamine-pre-contracted domestic pig CA rings (Fig 1E and 1F, 16.6±5.6% of dilation in present of XE991). This indicates that ML213 dilates histamine-pre-contracted CA rings by enhancing the activity of functional K_v_7 channels.

While performing these experiments, we observed that histamine-induced contractions were not sustained in domestic CA rings. After reaching a peak, the contractile force rapidly declined to a mean level of 31.2±5.5% at 4 minutes of the peak value (Fig 1C, 1D-“His-a,” 1E-“His-c,” and 1J-“His-e”) despite the continuous presence of histamine in the tissue bath. Subsequent applications of histamine caused contraction with a decreased strength (Fig 1D-“His-b” and 1J-“His-f”) even after a 20 minute wash with the standard Krebs buffer. On the other hand, KCl-induced contractions were sustained and had similar amplitudes over the course of the entire experiment (Fig 1J), indicating that the ring’s “health” was not compromised. With time, the sensitivity of the rings to histamine slowly recovered, but this required about an hour. Surprisingly, we found that the histamine-pretreated domestic pig rings exhibited an immediate recovery from the histamine-induced tachyphylaxis in the presence of XE991 (Fig 1E-“His-d” and 1J-“His-g”). In addition, we observed that the histamine-induced contractile force decayed much more gradually to a mean level of 3.47±1.28% at 4 min in the presence of XE991 (Fig 1E-“His-d” and 1J-“His-g”). For instance, during the experiment in Fig 1J, the contractile force decreased by 95% over a period of 8 minutes (“His-e”) in the presence of histamine, but only decayed by 14.9% after 8 minutes in “His-g” when XE991 was present in the bath with histamine. Nifedipine fully inhibited the sustained histamine-induced contractions observed in the presence of XE991, indicating that Ca^2+^ influx through the voltage-gated Ca^2+^ channels is accountable for the sustained contractions. Thus, the apparent histamine-induced tachyphylaxis was less pronounced in the rings with a reduced functional activity of K_v_7 channels, suggesting that the K_v_7-dependent hyperpolarization may be responsible for facilitating the kinetics of decay of histamine-induced contractions.

### K_v_7.4 activity likely contributes more to modulating histamine-induced contractions

**Fig 2:**
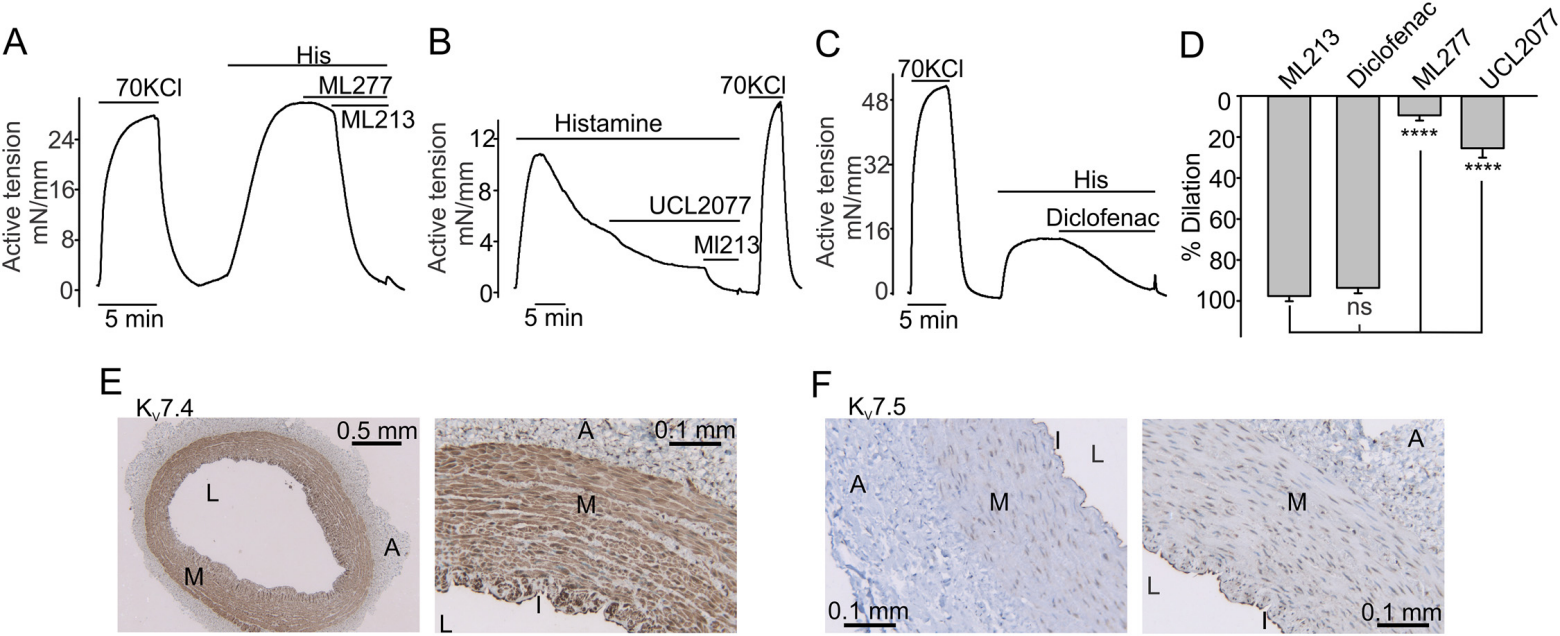
Immunostaining of K_v_7 channels in the CA. **A**, 10 μM ML277, an opener of K_v_7.1 channels, did not affect the tension in histamine-pre-contracted domestic pig CA rings. A sample trace is shown. **B and C**, Shown are sample isometric tension recordings illustrating that a K_v_7.4 activator diclofenac (200 μM) causes greater dilations of histamine-pre-contracted rings than a K_v_7.5 activator UCL2077 (50 μM). **D**, Summary data for “A-C” (ML213: n = 12; Diclofenac: n = 5; ML277, n = 8; UCL2077: n = 5). In these experiments, we selected those domestic pig CA rings that exhibited the slower decay rate of histamine-induced contractions. **E and F,** Images illustrating typical immunohistochemical staining patterns for K_v_7.4 and K_v_7.5 isoforms in the domestic pig CA wall.

Previously, K_v_7.1, K_v_7.4, and K_v_7.5 proteins were detected in the domestic pig coronary arteries using either PCR or Western blotting techniques [31]. We utilized a pharmacological approach to identify the isoform(s) of K_v_7 channels involved in regulating the histamine-induced contraction shape in the domestic pig CA. We first tested whether a K_v_7.1 activator ML277 dilates histamine-pre-contracted domestic CA rings. We found that ML277 did not significantly affect the tension in the histamine-pre-contracted CA rings (Fig 2A), whereas ML213 potently dilated the same rings. This suggests the contribution of K_v_7.1 in regulating histamine-induced contractions is negligible in domestic pig CAs.

We described above that ML213 potently dilates the domestic pig histamine-pre-contracted CAs. ML213 is known as an activator of K_v_7.2, K_v_7.4, and K_v_7.5 channels [32,33]. Since K_v_7.2 were not detected in domestic pig CAs [31], we hypothesized that either K_v_7.4 or K_v_7.5 activation may result in dilation of histamine-pre-contracted domestic pig CAs. To identify which of these two channels are more functionally important for regulating domestic pig CA histamine-induced contractions, we employed two additional modulators of K_v_7.4 and K_v_7.5 channels, UCL2077 and diclofenac. UCL2077 was previously reported to weakly block K_v_7.4 and potently potentiate K_v_7.5 channels [34], whereas diclofenac was shown to inhibit the K_v_7.5 channel and potently activate the K_v_7.4 channel [35]. We observed that 50 μM UCL2077 induced weak dilations (Fig 2B and 2D), whereas diclofenac (200 μM) caused potent dilations in the histamine-pre-contracted domestic pig CA rings (n = 5, Fig 2C and 2D), supporting the hypothesis that the K_v_7.4 channel may be more important for modulating histamine-induced contractions in the domestic pig CAs than the K_v_7.5 channel.

### Distribution pattern of K_v_7.4 and K_v_7.5 proteins in the wall of domestic pig CAs

We next used the immunohistochemistry approach to determine K_v_7.4 and K_v_7.5 protein distribution patterns within the wall of domestic pig CAs. We observed a strong K_v_7.4 immunostaining in the medial and intimal layers (Fig 2E). Conversely, K_v_7.5 immunostaining was strong only in the intimal layer (I, endothelium), with the medial layer (M, smooth muscle cell layer) exhibiting much weaker K_v_7.5 staining intensity (Fig 2F) in the CA sections probed with the specific K_v_7.5 antibody [30]. Only scattered K_v_7.4 and K_v_7.5 staining was observed in the adventitia (Fig 2E and 2F). These data are consistent with our pharmacological results obtained during the isometric tension measurements.

### ML213 weakly dilates KCl-pre-contracted rings

**Fig 3:**
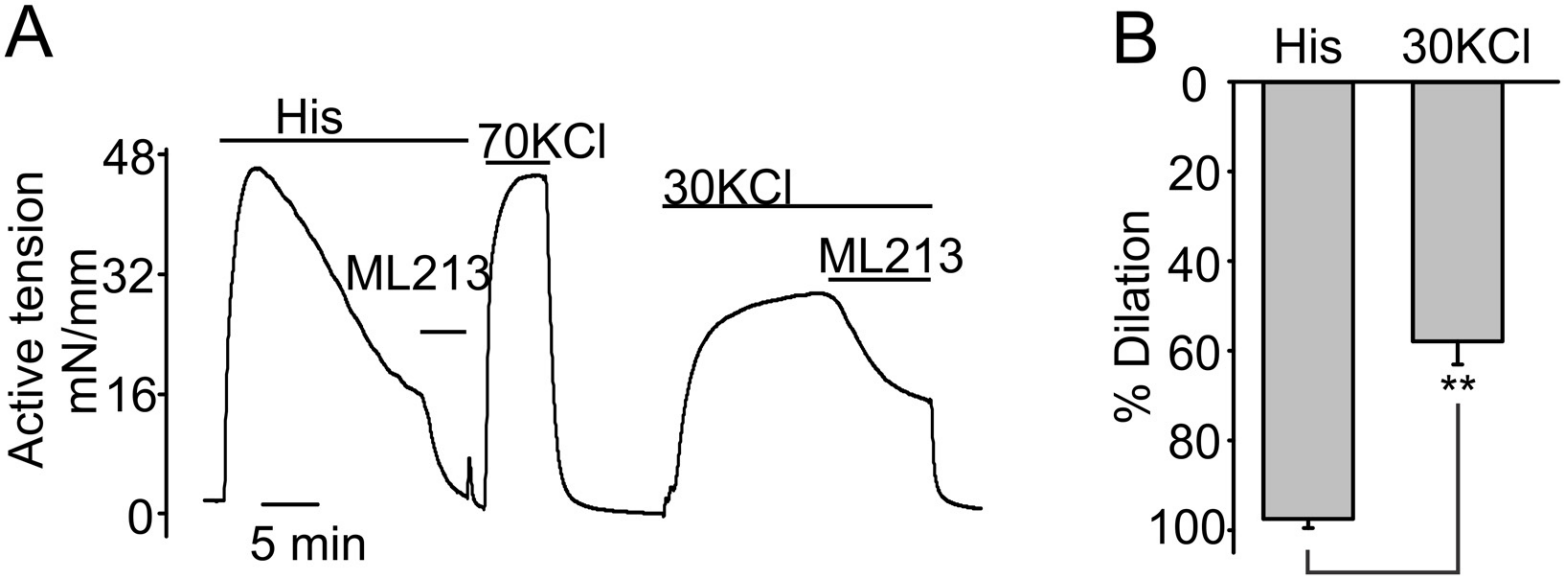
Effects of ML213 on KCl-pre-contracted CAs. **A,** Sample isometric tension recoding illustrating the effects of 10 μM ML213 on the rings pre-constricted with histamine or 30 mM KCl. **B**, Summary data for ML213 effects in pre-contracted CA rings.

We next assessed whether ML213 modulates the KCl-induced contractions in the domestic pig CAs. ML213 only partially dilated the 30 mM KCl-pre-contracted CA rings (57.9±5.2% relaxation, n = 4, Fig 3A and 3B). These results suggest that the efficacy of ML213 for relaxing CA rings decreases at the higher concentrations of bath KCl. This is most likely due to the fact that the equilibrium Nernst potential for potassium is shifted towards a more positive value at a higher bath concentration of potassium, limiting the window of potentials at which potassium efflux repolarizes smooth muscles.

### Functional role of K_v_7 channels in the intimal layer

**Fig 4:**
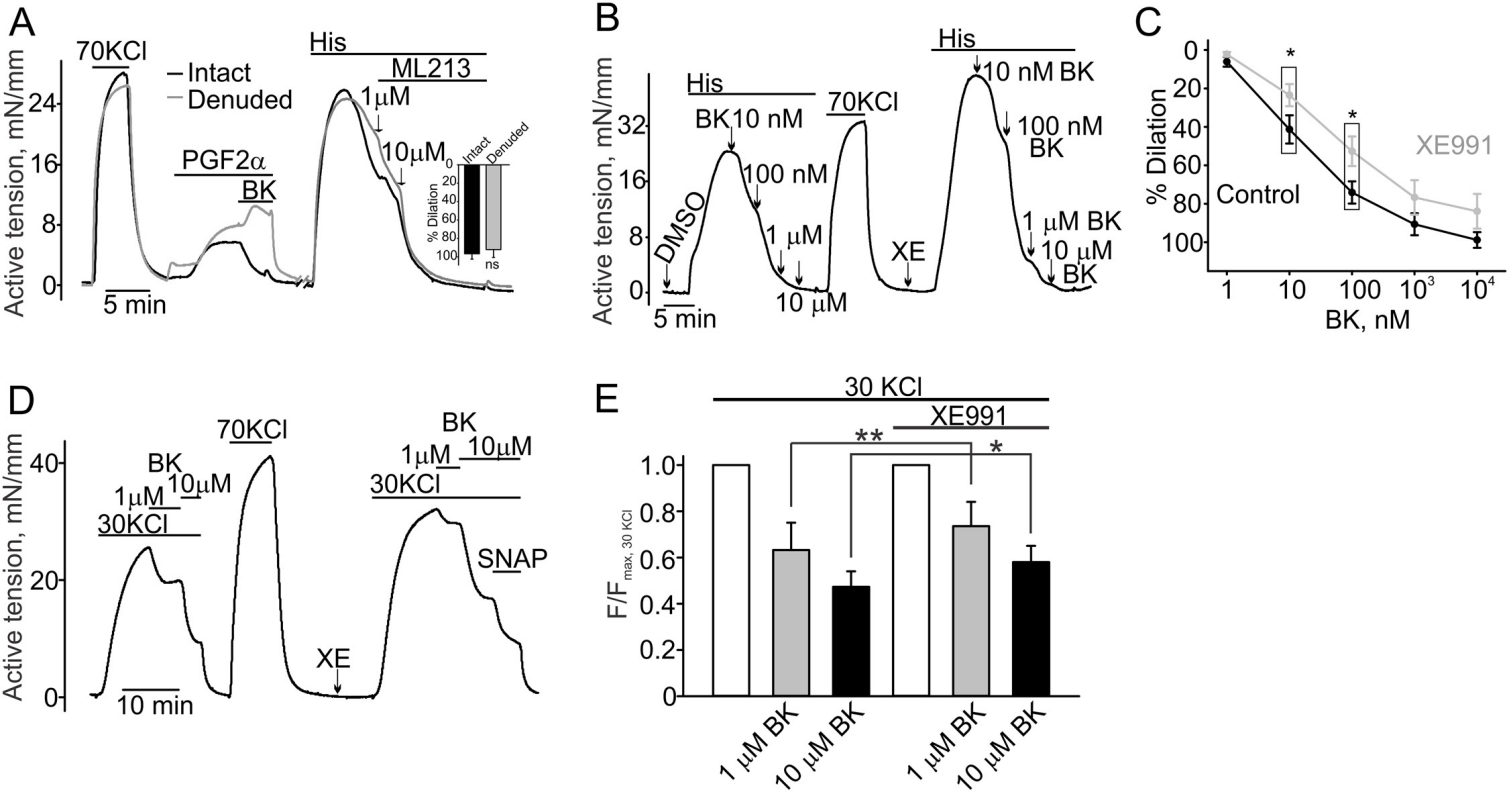
Functional role of K_v_7 channels in the endothelium. **A,** Sample isometric tension measurement traces obtained in intact (black trace) and denuded (grey trace) domestic pig CA rings are shown. The insert shows that both intact and endothelium-denuded CA rings were equally dilated by ML213 (10 μM, n = 3). PGF_2α_ stands for Prostaglandin F_2α_ (10 μM). **B,** A sample trace illustrating that the bradykinin-induced dilation is reduced in the presence of XE991 (10 μM) in a histamine-pre-contracted CA ring (10 nM bradykinin: 41.3±7.3% for DMSO vs. 23.5±5.73% for XE991; 100 nM bradykinin: 74.1±5. 8% for DMSO vs. 52.7±7.7% for XE991, n = 8). **C**, The concentration-response curves for bradykinin-induced dilations of histamine-pre-contracted rings obtained in the presence or absence of XE991 (10 μM). **D**, A sample trace illustrating the effect of XE991 (10 μM) on bradykinin-induced dilations in a 30 mM KCl-pre-contracted RCA ring. XE991 reduced bradykinin-induced relaxations. **E**, The summary for the data shown in the panel D. BK stands for bradykinin. Coronary artery rings were treated with 1 μM BK (n = 6) and 10 μM BK (n = 7).

Our immunohistochemistry data revealed that both K_v_7.4 and K_v_7.5 proteins are highly expressed in the CA intimal layer (Fig 2E and 2F) of domestic pig CAs. The intimal layer is made of mainly endothelial cells, suggesting that K_v_7 channels may contribute to regulating the endothelial function in CAs. A major function of the endothelial cells is to produce vasodilatory nitric oxide (NO). To investigate whether endothelial NO production underscores ML213-induced dilations in histamine-pre-contracted rings, we mechanically removed the endothelial layer in CAs and investigated the ML213 effect on the denuded histamine-pre-contracted domestic CAs. We found that ML213 still potently dilated the mechanically denuded histamine-pre-contracted CA rings (Fig 4A). Thus, the activation of K_v_7 channels expressed in smooth muscles, but not in the endothelium, is critical for mediating ML213-dependent CA dilations.

The activation of the endothelial K_v_7 channels should hyperpolarize the endothelial cells. This in turn may potentiate hormone-stimulated NO production in the CAs because endothelial cell hyperpolarization should favor Ca^2+^ influx through endothelial receptor- and store-operated channels. Therefore, we explored whether endothelial bradykinin-induced NO production is affected in the presence of XE991. We found that in the presence of XE991, the magnitude of 10 nM and 100 nM bradykinin-induced dilations were significantly smaller in histamine-pre-contracted domestic pig CA rings compared to those observed in the presence of the vehicle control (Fig 4B and 4C). Higher concentrations of bradykinin (>100 nM) almost completely dilated histamine-pre-contracted rings; and the bradykinin-dependent dilations were not significantly different in the DMSO and XE991 groups. To confirm that bradykinin induces nitric oxide production in domestic pig CAs, we used an eNOS inhibitor N^g^-Nitro-L-arginine Methyl Ester (N^g^NLA, 100 μM) that completely eliminated bradykinin-induced dilations (S2 Fig).

To exclude the possibility that the observed XE991-dependent decreases of bradykinin-induced dilations are strictly related to histamine pretreatments, we investigated whether XE991 modulates the bradykinin-induced dilations in the 30 mM KCl-pre-contracted CA rings. 10 μM XE991 again significantly reduced bradykinin-induced dilations in KCl pre-contracted rings (Fig 4D and 4E). In this experiment, we used an NO donor, S-Nitroso-N-Acetyl-D,L-Penicillamine (SNAP), to test if the bradykinin-dilated rings can be further relaxed by an additional bolus of free NO in the presence of 10 μM XE991. This experiment suggests that XE991 pretreatment is unlikely to affect the ability of vascular smooth muscles to be relaxed by free NO. Thus, the observed smaller bradykinin-induced dilations in the presence of XE991 rather indicate the reduced NO production by the coronary endothelium.

### K_v_7.4 immunostaining and function in MetS Ossabaw pig CAs

We next assessed the contribution of K_v_7 channels in regulating the coronary reactivity in MetS CAs. In these experiments, we used a miniature Ossabaw pig model of MetS. Ossabaw pigs display all of the characteristics of MetS, including hypertension, insulin resistance, dyslipidemia, obesity, and glucose intolerance, when fed an atherogenic diet for about 7–10 months [23] starting from an age of 6–12 months.

**Fig 5:**
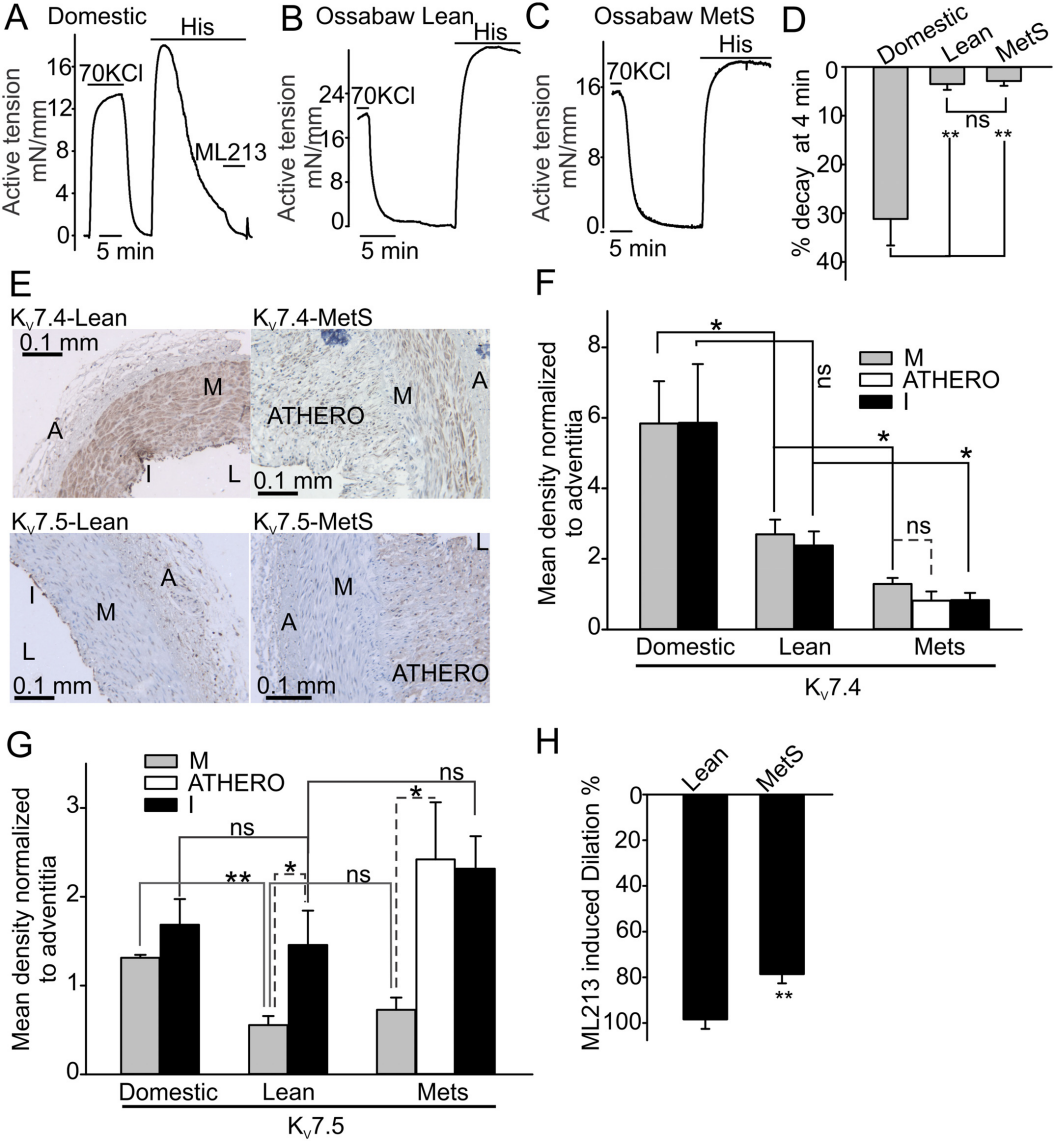
K_v_7.4 and K_v_7.5 immunostaining and function in Ossabaw pig CAs. **A-D**, Sample isometric tension recordings and summary data illustrating different decay rates of histamine-induced contractions observed in domestic and Ossabaw CA rings (Domestic: n = 7; Lean: n = 7; MetS: n = 5). **E** shows the pattern of K_v_7.4 and K_v_7.5 protein distribution across the conduit coronary artery cross-sectional segments from Ossabaw pigs. **F and G**, Summary data for K_v_7.4 and K_v_7.5 protein distribution. The ratio of the background-corrected intensities of media-intimal layer and the adventitia layer staining was calculated to quantify the relative expression levels of the K_v_7 proteins (Domestic: n = 3; Lean: n = 4; MetS: n = 3). **H** shows the summary data illustrating 10 μM ML213 dilatory effects in histamine pre-contracted rings from Ossabaw lean and MetS pigs (Lean: n = 5; MetS: n = 6).

Histamine induced potent contractions in both control lean and MetS Ossabaw pig CAs. Surprisingly, we found that the histamine-induced contractions were sustained and decayed only by 3.5±1.2% and 2.9±1.0%, respectively within 4 minutes after histamine application (Fig 5B–5D, compare to domestic pig CA in Fig 5A). Based on our previous finding that XE991 slows the decay rate of histamine-induced contractions in domestic pig CAs, we hypothesized that a higher level of expression or activity of K_v_7.4 or K_v_7.5 channels may underlie the transient nature of histamine-induced responses, whereas reduced K_v_7 expression would be associated with more sustained contractions.

To test this hypothesis, we evaluated the relative expression of Kv7 proteins in Ossabaw pig CAs by using the immunohistochemistry approach. Similarly to domestic pig CAs, the K_v_7.4 staining was clearly visible in the medial and intimal layers of lean Ossabaw pig CAs (Fig 5E). However, the intensity of the staining was almost two times smaller as compared to that in the domestic pig CA sections (Fig 5F). In lean Ossabaw CA sections, the K_v_7.5 immunostaining was very weak in the smooth muscle medial layer and strong in the endothelium (Fig 5F and 5G). Some K_v_7.5 immunostaining was also detected in the adventitia (Fig 5E).

We then determined the protein expression pattern of K_v_7.4 and K_v_7.5 proteins in CA wall segments from the MetS Ossabaw pigs. We found that K_v_7.5 staining was barely detectable in the medial smooth muscle layer of MetS CAs, but strong in in the intima layer and adventitia (Fig 5E). The K_v_7.4 immunostaining was clearly seen in the medial layer of MetS Ossabaw pig CAs. However, it was also about twice as weak as in the lean Ossabaw pig CAs (Fig 5F, 2.69±0.42 vs 1.38±0.22, p<0.05) and four times weaker than in the domestic pig CA (Fig 5F, 5.84±1.2 vs 1.38±0.22, p<0.05, respectively). Unexpectedly, we found that the atheroma segments of the MetS CAs expressed relatively higher levels of K_v_7.5 proteins compared to the medial layer. K_v_7.4 was also present in the proliferating smooth muscle cells of the atheroma, but its expression was comparable to that in the medial layer. Since the atheroma segments do not contribute to contraction force generation in CAs, these observations were not followed up in the present study. Thus, our immunohistochemistry results suggest that a lower decay rate of histamine-induced contractions observed in the Ossabaw pig CA rings may likely be associated with a weaker medial K_v_7 immunostaining intensity. To further verify that the medial K_v_7 immunostaining intensity correlates with functional activity of the channels in the Ossabaw pig CAs, we compared the ability of ML213 to dilate lean and MetS Ossabaw CA rings pre-contracted with histamine. We found that MetS CAs, expressing a reduced level of Kv7 proteins, exhibited smaller 10 μM ML213-induced dilations in the histamine-pre-contracted rings as compared to those in lean Ossabaw CA rings (Fig 5H).

## Discussion

The main new findings of this study are as follows. 1) We describe the coronary wall distribution of K_v_7.4 and K_v_7.5 proteins in the “healthy” and MetS pig right coronary arteries; 2) We demonstrate that K_v_7.4 and K_v_7.5 channels are likely inactive in the preloaded pig right coronary arteries; therefore, the channels’ inhibition by histamine is unlikely to be the major contributing factor in triggering histamine-induced coronary contractions in the pig right coronary arteries; 3) We ascertain that ML213 dilates the histamine-pre-contracted pig right coronary arteries in an endothelium-independent manner, likely by activating K_v_7.4 and K_v_7.5 channels expressed in the smooth muscles of the pig right coronary artery; 4) We reveal that K_v_7.4 and K_v_7.5 channel activity is critical for augmenting bradykinin-induced dilations of histamine- or KCl-pre-contracted rings in a endothelium-dependent manner. 5) We provide evidence that the decay rate of histamine-induced contractions inversely proportionally correlates with the level of K_v_7 activity and smooth muscle K_v_7.4 and K_v_7.5 immunostaining intensity.

A recent study by Morales-Cano et al. [30] provided strong evidence that K_v_7 channels are differentially expressed in the right and left rat coronary arteries, with the K_v_7.4 expression level being 2–3 fold greater in right rat coronary arteries compared to that of K_v_7.1 and K_v_7.5. Using pharmacological techniques, we ruled out the involvement of K_v_7.1 channels in regulating histamine-induced contractions in domestic pig right coronary arteries. However, our pharmacological data confirm that there may be a greater contribution of K_v_7.4 to regulating the histamine-induced contractions as compared to that of K_v_7.5 in pig right coronary artery. That is consistent with the Morales-Cano et al. results.

Morales-Cano et al. [30] demonstrated that XE991 weakly contracts the resting rat right coronary arteries only at the concentrations exceeding 0.3 μM. In contrast, much smaller XE991 concentrations (0.03 μM) caused contractions in the rat left coronary artery. The authors attributed such a difference in the XE991 sensitivity between the left and right coronary arteries to a higher level of expression of K_v_7.5 in the left coronary artery. Indeed, potassium channels in coronary artery smooth muscle cells contribute to setting the resting membrane potential, with K_v_7.4 exhibiting a more positive threshold of activation than K_v_7.5 (-50 mV for K_v_7.4 [27] versus -62 mV for K_v_7.5 [29]. This means that K_v_7.4 channels are more likely to be inactive at the resting membrane potential of about -60 mV in smooth muscle cells. Therefore, the lack of XE991-induced contractions may be consistent with a higher K_v_7.4 expression and lower K_v_7.5 expression in coronary arteries.

Our data indicate that 10 μM XE991 does not induce any significant contractions in the domestic pig right coronary artery. On the other hand, consistently with the Morales-Cano et al. study, we also find that XE991 weakly contract the pig left coronary arteries (about 27.3% of histamine-induced contractions, S3 Fig). These results indicate that the histamine-dependent inhibition of K_v_7 is unlikely to contribute significantly to triggering histamine-induced contractions in the pig right coronary artery. But, that may be a factor promoting histamine-induced contractions in the left coronary arteries. Insensitivity of the pig right coronary artery to XE991 also suggests that the artery may exhibit a relatively lower K_v_7.5 expression and a relatively higher K_v_7.4 expression, which is in good agreement with our pharmacological results. Thus, depending on the relative expression of K_v_7.4 and K_v_7.5 channels as well as the membrane potential in smooth muscle cells, the inhibition of K_v_7 channels either by small molecule blockers or via the receptor-mediated pathway may variably affect the pig coronary tone.

It has been demonstrated that metabolic syndrome significantly impairs the control of coronary blood flow [36–38] in part due to K^+^ channel dysregulation. For example, the penitrem A-sensitive and NS1619-activated BK_Ca_ current was reduced in coronary artery smooth muscle cells from MetS Ossabaw pigs [39]. Since MetS is considered as a pre-diabetic condition, it is interesting to note that the function of K_v_7 channels was reduced in diabetic rat coronary arteries [40,41]. Consistently, we also find that K_v_7 immunostaining intensity is significantly weaker in MetS pig right coronary arteries as compared to the lean control pig right coronary artery.

Interestingly, our data indicate that the proliferating smooth muscle cells in the MetS atherosclerotic segments of pig right coronary arteries exhibit an increased expression of K_v_7.5. It is known that only medial layer smooth muscle cells contribute to force generation in coronary arteries [42,43], whereas the proliferating smooth muscle cells found in the atheroma segments do not contract. This suggests that during coronary atherosclerosis progression, the transformation of the smooth muscle cells from the contractile phenotype to the proliferative non contractile phenotype is accompanied by a relative increase of K_v_7.5 expression. Conversely, the K_v_7.4 expression level in proliferating smooth muscle cells of atheromas was comparable to that observed in the contractile smooth muscle cells of the medial layer. Currently, the functional implications and the mechanisms underlying such an earlier molecular switch that may lead to the alteration in K_v_7.5 expression pattern occurring during the progression of atherosclerosis remains unclear.

**Fig 6:**
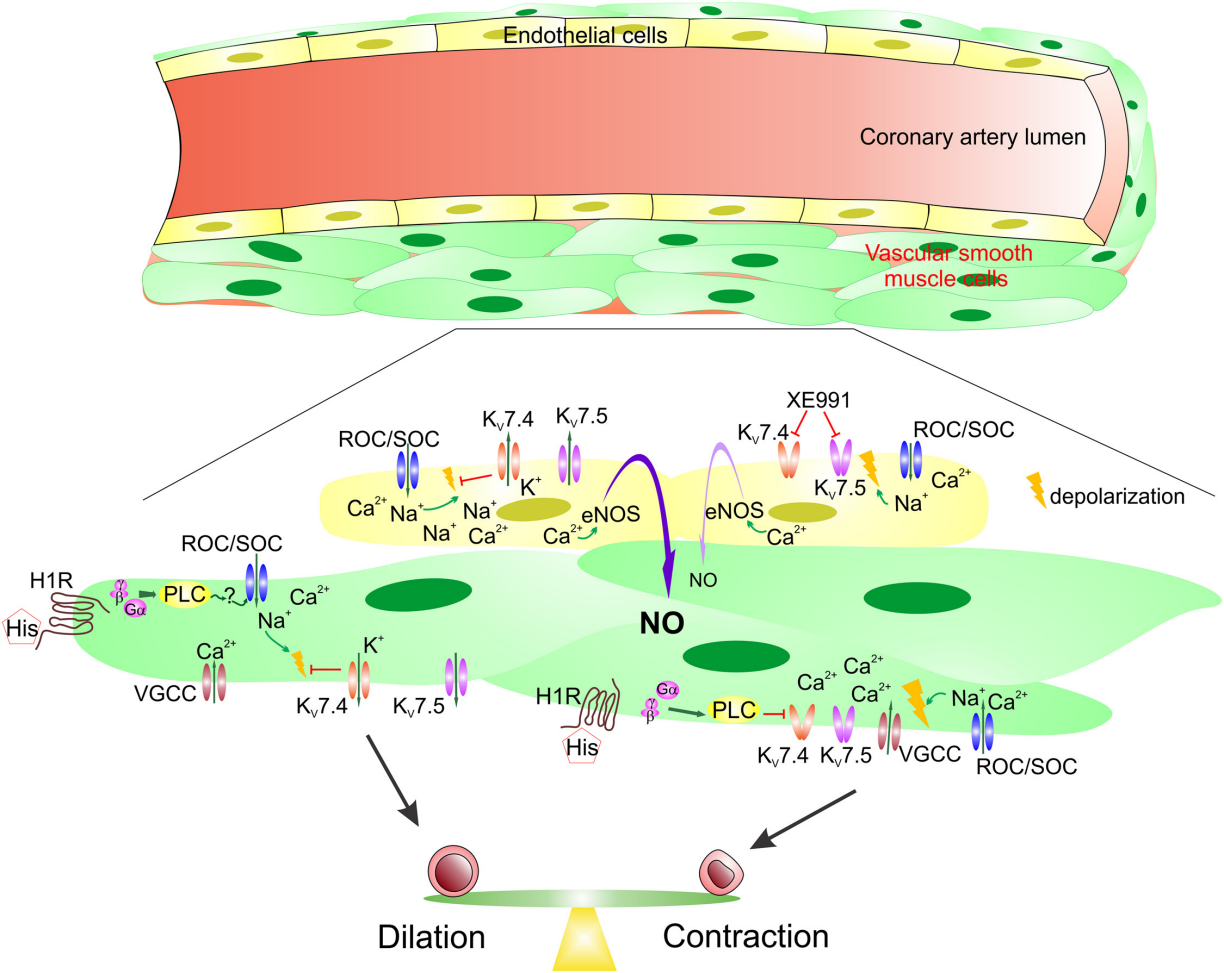
Diagram summarizing the roles of K_v_7 channels in the pig right coronary artery. It appears that a balance between K_v_7 channel activation and inhibition contributes to sculpturing the shape of histamine-induced CA contractile responses. Histamine acts on the histamine H1 receptor (H1R) expressed in CA smooth muscle cells. This results in a phospholipase C (PLC)-dependent stimulation of receptor-operated and store-operated channels (ROC/SOC) mediating cation influx that depolarizes the smooth muscle cells. In turn, smooth muscle cell depolarization activates voltage-gated Ca^2+^ channels that leads to massive Ca^2+^ influx into the smooth muscle cells with a consecutive smooth muscle contraction and further depolarization. Such stronger depolarization stimulates K_v_7 activity repolarizing smooth muscles and weakening coronary artery ring contractions. Conversely, histamine-dependent inhibition of K_v_7 channels via a PLC-dependent mechanism leads to an increased smooth muscle cell depolarization and strengthens ring contractions. In endothelium, bradykinin-dependent ROC/SOC activation also results in Na^+^ and Ca^2+^ influx that is driven by the gradient of these cations and negative potential inside endothelial cells. However, such cation influx depolarizes endothelial cells reducing the driving force. Activation of endothelial K_v_7 channels likely enhances endothelium-dependent dilatory responses in the pig right coronary artery by maintaining more negative potential inside endothelial cells that results in greater Ca^2+^ influx known to stimulate Ca^2+^-dependent nitric oxide synthase (eNOS) causing increased NO production.

We indicated above that histamine-dependent inhibition of the pre-activated K_v_7 channels does not seem to be the major mechanism for triggering the histamine-induced contractions at least in pig right coronary arteries. But what then is the trigger? It has been shown that G_q/11_-protein-coupled receptor agonists activate the receptor-/store-operated cation influx (ROC/SOC, [44]), leading to depolarization of the artery smooth muscles. The ROC/SOC-dependent depolarization activates smooth muscle voltage-gated Ca^2+^ channels mediating massive Ca^2+^ influx which results in the smooth muscle contraction. Ca^2+^ influx would also further depolarize the smooth muscles. We propose that a similar mechanism underlies the smooth muscle contractions in domestic pig right coronary arteries (Fig 6). However, we speculate that a weaker ROC/SOC-dependent depolarization is not sufficient for activating K_v_7s. It appears that only more strong depolarization associated with the activity of voltage-gated Ca^2+^ channels is required to stimulate K_v_7 activity. Once activated, K_v_7s mediate potassium efflux which repolarizes smooth muscles causing the histamine-induced contraction force decay. Possibly, the histamine-dependent inhibition of K_v_7s leads to a reduced beneficial hyperpolarizing action of the activated K_v_7 channels, prolonging histamine-induced contractile responses (Fig 6). Importantly, it appears that K_v_7.4 and K_v_7.5 channel activator ML213 is capable of opening even the histamine-inhibited K_v_7.4 channel, providing a wide horizon for the compound’s usage as an antispasmodic drug to treat coronary spasm.

Blood plasma histamine levels are higher in the patients presenting with acute coronary syndrome, a complication of MetS [6,45]. Our finding that K_v_7 channel expression is smaller in the coronary medial layer of MetS pigs supports the hypothesis that weakening the balance arm responsible for hyperpolarizing coronary artery smooth muscle cells shifts the balance towards the spasmodic state (Fig 6). Endothelial dysfunction, a feature of MetS, further shifts the balance towards the increased spasticity. We report here that the inhibition of K_v_7 activity decreases endothelium-dependent relaxation in intact coronary arteries. Thus, inhibitors of K_v_7s may pharmacologically cause endothelial dysfunction in coronary arteries, further promoting spasticity. We speculate that endothelial K_v_7-dependent hyperpolarization may favor endothelial NO production, likely by promoting Ca^2+^ influx through endothelial bradykinin-activated ROCs/SOCs. Thus, stimulation of K_v_7 activity might be a promising way for remediating endothelial dysfunction.

In conclusion, our findings reveal that the activity of K_v_7.4 and K_v_7.5 play a complex role in modulating the shape and cumulative strength of histamine-induced contractions in the pig right coronary artery. It appears that smooth muscle K_v_7 activation is important for modulating the decay rate of the histamine-induced contractions, whereas endothelial K_v_7 activation plays a role in potentiating bradykinin-induced endothelium-dependent dilations in pre-contracted right coronary arteries. Both effects lead to reduced spasticity. We found that MetS right coronary arteries exhibit a reduced expression of K_v_7 channels that is associated with sustained histamine-induced contractions in the model. Importantly, our data suggest that ML213 is capable of potently dilating the strongest histamine-induced contractions of the MetS coronary arteries. Thus, ML213 and probably other K_v_7.4 and K_v_7.5 selective activators may be useful for preventing or reducing coronary spasm (angina pectoris episodes) in the patients presenting with elevated plasma histamine concentrations due to mast cell atheroma infiltration or during acute allergic reaction associated with plasma histamine surges.

## Supporting Information

S1 Fig Light microscopy immunohistochemical images of pig RCA sections. The sections were probed and developed as described in the Methods’ Immunohistochemistry protocol with the exception that the primary antibodies were not added.
(TIF)

S2 Fig Bradykinin-dependent dilations are nitric oxide dependent in pig CAs. **A and B** show sample isometric tension traces, illustrating that bradykinin-induced dilations are not observed in Ossabaw (**A**) and domestic (**B**) pig CA rings pretreated with an eNOS inhibitor, N^g^-Nitro-L-arginine Methyl Ester (N^g^NLA, 100 μM, n = 4). BK stands for bradykinin (10 μM).
(TIF)

S3 Fig Effect of XE991 on basal tone in domestic pig coronary arteries. **A** shows the two sample traces illustrating the effects of 50 μM histamine, 70 mM KCl and 10 μM XE991 in the domestic pig right (solid line, RCA) and left (broken line, LCA) coronary arteries. **B**. Summary data for 10 μM XE991-induced contraction amplitudes in resting preloaded coronary artery rings compared to histamine- and KCl-induced contraction amplitudes.
(TIF)
