# Supplementary figures and images for: Novel Roles for Kv7 Channels in Shaping Histamine-Induced Contractions and Bradykinin-Dependent Relaxations in Pig Coronary Arteries
Source: PLoS One. 2016 Feb 4;11(2):e0148569. doi: 10.1371/journal.pone.0148569 (PMC4742238; doi:10.1371/journal.pone.0148569)

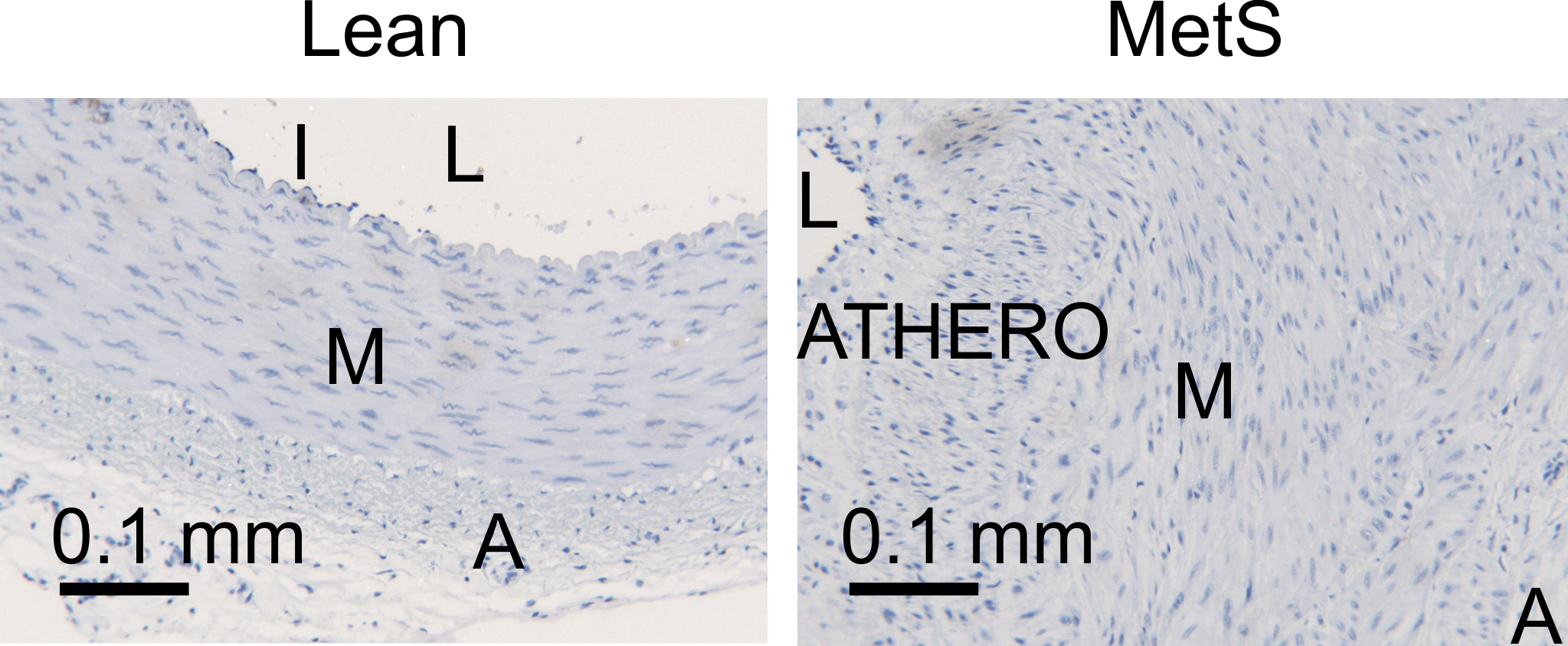

Supplement: S1 Fig — The sections were probed and developed as described in the Methods’ Immunohistochemistry protocol with the exception that the primary antibodies were not added. (TIF) [file pone.0148569.s001.tif]

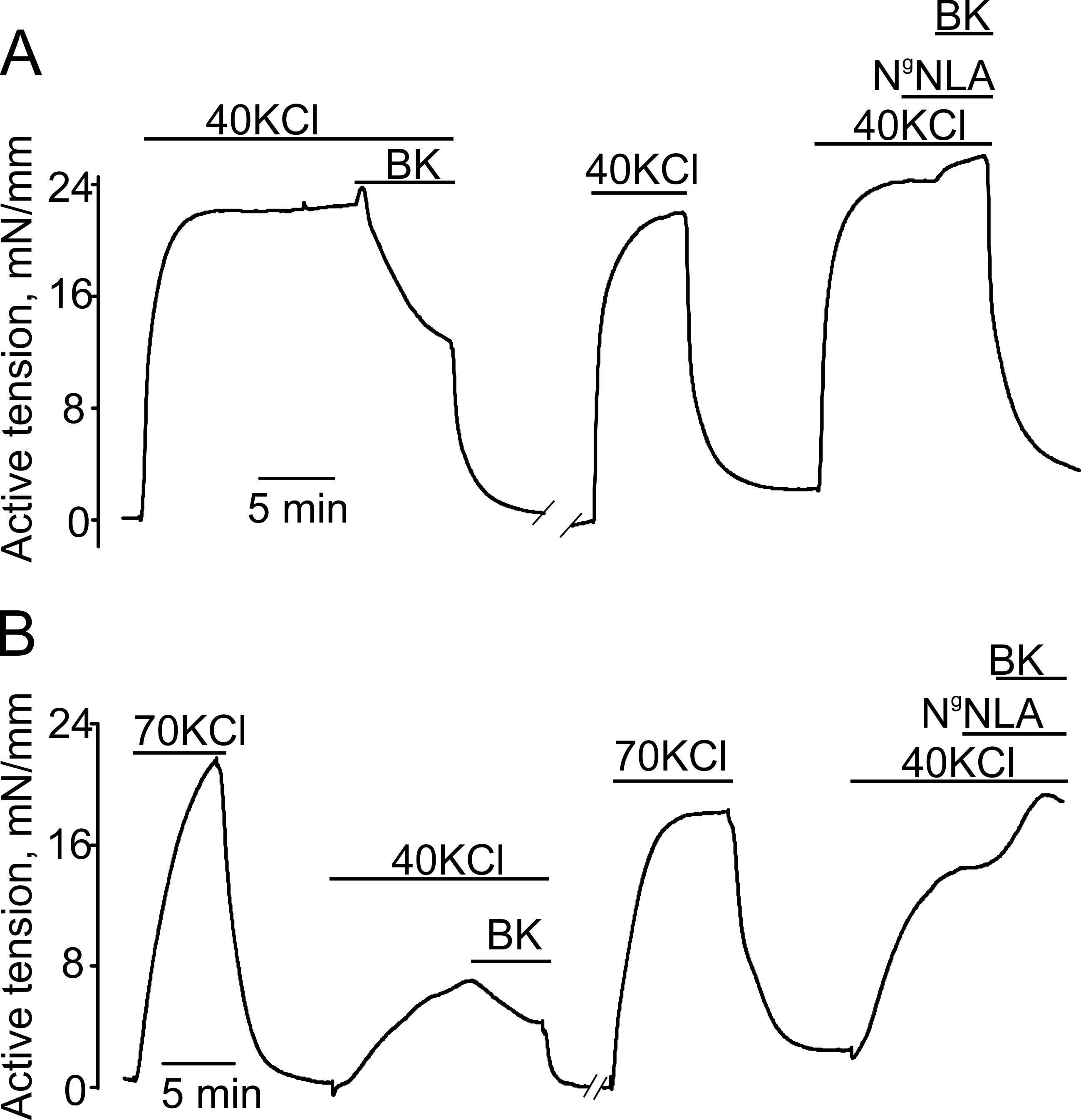

Supplement: S2 Fig — A and B show sample isometric tension traces, illustrating that bradykinin-induced dilations are not observed in Ossabaw (A) and domestic (B) pig CA rings pretreated with an eNOS inhibitor, Ng-Nitro-L-arginine Methyl Ester (NgNLA, 100 μM, n = 4). BK stands for bradykinin (10 μM). (TIF) [file pone.0148569.s002.tif]

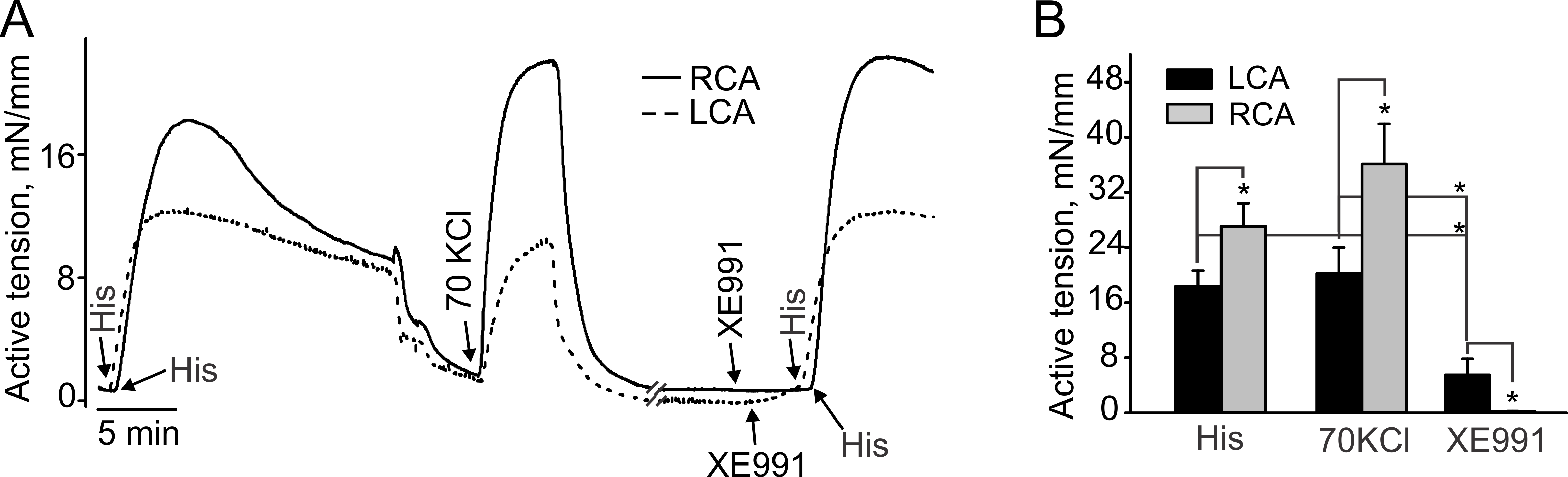

Supplement: S3 Fig — A shows the two sample traces illustrating the effects of 50 μM histamine, 70 mM KCl and 10 μM XE991 in the domestic pig right (solid line, RCA) and left (broken line, LCA) coronary arteries. B. Summary data for 10 μM XE991-induced contraction amplitudes in resting preloaded coronary artery rings compared to histamine- and KCl-induced contraction amplitudes. (TIF) [file pone.0148569.s003.tif]
